# Ebola Virus Entry: A Curious and Complex Series of Events

**DOI:** 10.1371/journal.ppat.1004731

**Published:** 2015-04-30

**Authors:** Sven Moller-Tank, Wendy Maury

**Affiliations:** 1 Department of Microbiology, University of Iowa, Iowa City, Iowa, United States of America; 2 Gene Therapy Center, University of North Carolina at Chapel Hill, Chapel Hill, North Carolina, United States of America; University of Kentucky, Lexington, UNITED STATES

## Introduction

Ebola virus (EBOV) belongs to the *Filoviridae* family of negative-sense RNA viruses. Since its identification in 1976, sporadic outbreaks have occurred in Central Africa. The 2014 outbreak in West Africa provides evidence that EBOV is emerging into new geographic regions. While previous outbreaks have been confined to small areas, the most recent outbreak is atypically widespread with over 25,000 people infected. Evidence from the limited-sequence studies that have been published suggests that the outbreak resulted from a single reservoir-to-human transmission event and subsequent human-to-human spread [1]. As neither an EBOV vaccine nor antivirals are currently available, this outbreak highlights the critical need for the development of effective vaccines and therapeutics.

Infection is initiated by virions entering dendritic cells, macrophages, and, perhaps, hepatocytes [2]. Virus replication in these cells is thought to be critical for initiation of systemic infection, leading to virus spread to new sites with infection of additional cell populations. Thus, a better understanding of virus entry will not only provide insight into both host cell and virus biology, but also elucidate therapeutic targets. Here we provide a brief overview of the current understanding of EBOV entry and identify important questions that remain unanswered in the field.

## Filovirus Particles

**Fig 1:**
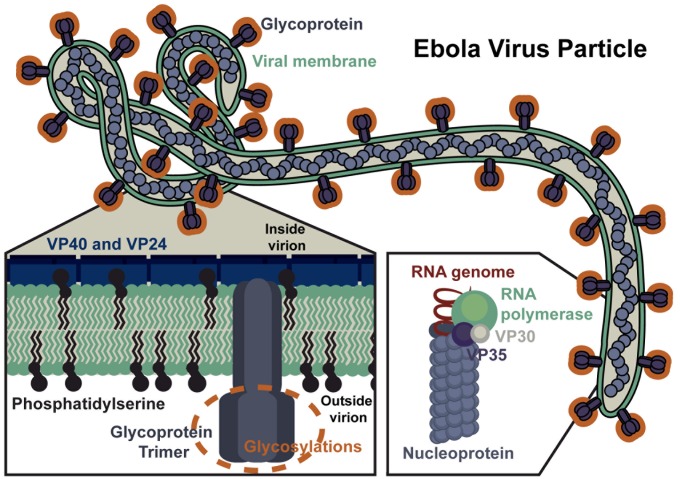
Ebola Virus Particle. An EBOV particle is shown with key viral proteins highlighted, including: the viral glycoprotein, matrix proteins (VP40, VP24), and viral ribonucleoprotein complex (RNA-dependent RNA polymerase, VP30, VP35, nucleoprotein, and RNA).

**Fig 2:**
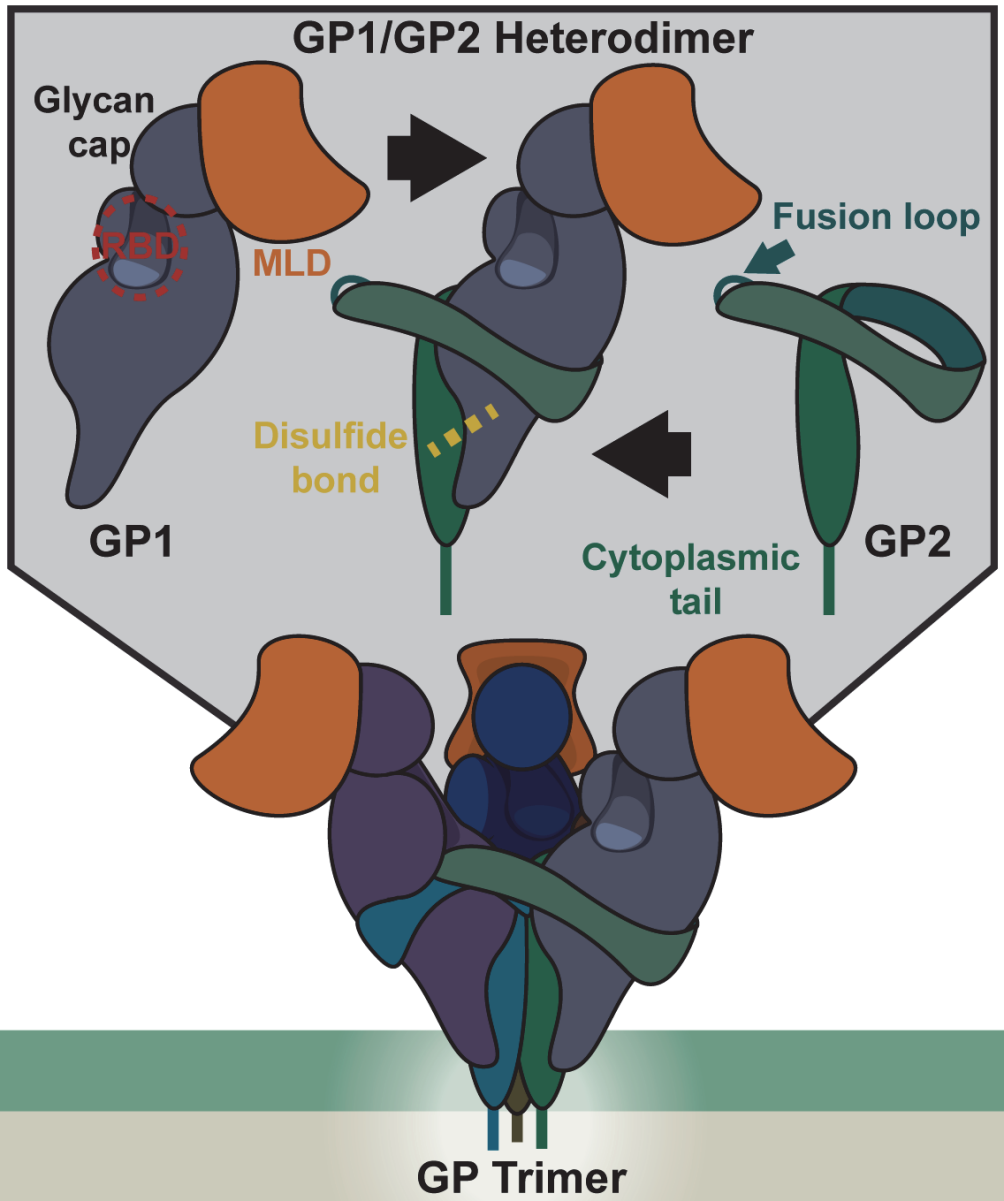
Structure of the EBOV glycoprotein (GP). (Top) Each monomer of GP consists of a GP1 and GP2 heterodimer associated via a disulfide bond. GP1 contains the receptor binding domain (RBD), protected by a glycan cap and a mucin like domain (MLD) at the apex of the structure. The RBD interacts with the endosomal receptor Niemann-Pick C1 (NPC1) upon proteolytic processing of GP1 that removes the glycan cap and MLD. The fusion loop, which imbeds in the target membrane during fusion, is part of the GP2 monomer, but remains hidden in the mature pre-fusion GP trimer. (Bottom)The GP trimer consists of three GP1/2 heterodimers (shown in different shades) that associate through several GP1/GP2 and GP2/GP2 interactions.

The uniquely shaped filamentous particles made by filoviruses are surrounded by an envelope acquired during virion budding from the plasma membrane (Fig 1). Recent studies provide evidence that the outer leaflet of the viral envelope contains phosphatidylserine (PtdSer), which serves as an important attachment factor during entry [3,4]. Inside the envelope, the viral matrix proteins VP40 and VP24 line the inner leaflet and provide structural support. Surrounded by this protective layer of lipids and matrix proteins, the RNA genome is associated with several viral proteins, forming the ribonucleoprotein (RNP) complex. A single viral glycoprotein (GP), encoded by the virus, embeds in the viral envelope and is required for virion/cellular membrane fusion. The mature GP is composed of two subunits, GP1 (~140 kDa) and GP2 (~26 kDa), that heterodimerize through disulfide bonds and associate to form trimers (Fig 2). The crystal structure of the EBOV GP reveals that this trimer forms a chalice-like shape, with the GP2 forming the base and GP1 forming the cup [5]. Surrounding and protecting this chalice is the N-glycan-containing cap region and a heavily N- and O-glycosylated mucin-like domain (MLD) of GP1. Glycans on these regions are important for shielding the GP from neutralizing antibodies [6].

## Adherence and Internalization

**Fig 3:**
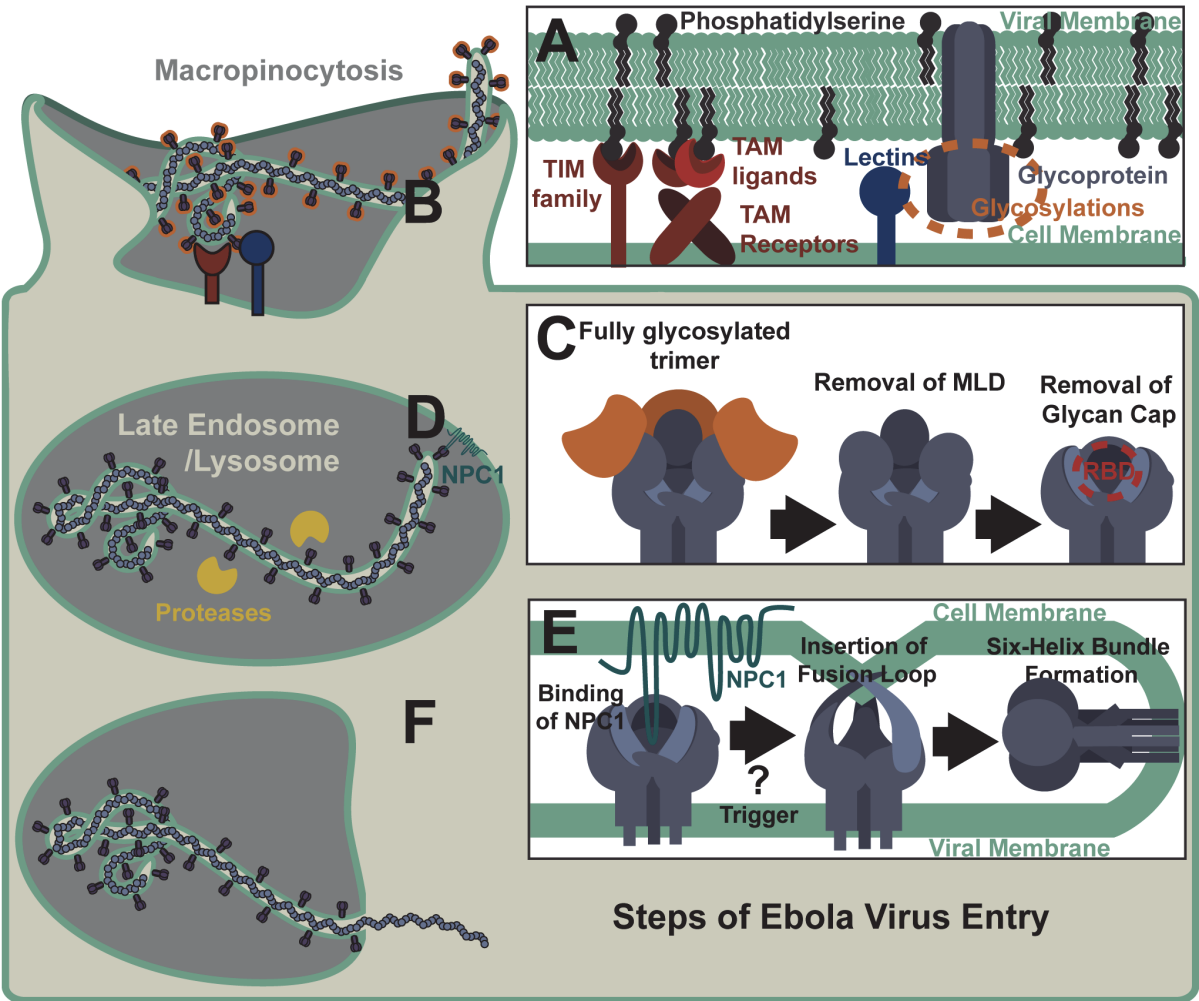
Steps of Ebola Virus Entry. (A) Cell surface receptors bind EBOV particles through interactions with either virion-associated phosphatidylserine or viral glycoprotein glycans. (B) Virus is internalized through ruffling of the plasma membrane and macropinocytosis. (C) During trafficking through endosomes, the EBOV glycoprotein is cleaved by proteases that remove the mucin-like domain (MLD) and glycan cap, exposing the receptor binding domain (RBD). Shown is a stepwise removal of those sequences, although in the cell these cleavage events may occur concurrently. (D) The RBD interacts with NPC1 in the late endosome/lysosome. (E) Binding of the NPC1 C-loop by the glycoprotein is followed by one or more triggers that release the fusion loop, allowing for its insertion into the target membrane. Subsequent transition of the EBOV GP into a six helix bundle results in the host and viral membranes being brought together, leading to fusion. (F) Release of the viral nucleoprotein into the cytoplasm prior to the initiation of virus replication.

The cell surface interactions of filoviruses differ from other characterized enveloped virus/cell surface receptor interactions in that amino acid residues of EBOV GP are not thought to interact with a cell surface receptor. Instead, these viruses bind to target cells through two types of relatively non-specific receptors: C-type lectins (CLECs) that interact with glycans on EBOV GP and PtdSer receptors that interact with the viral envelope PtdSer (Fig 3A). CLECs (LSECTin, DC-SIGN [dendritic cell-specific intercellular adhesion molecule-3-grabbing non-integrin], L-SIGN [liver/lymph node-specific ICAM-3 grabbing nonintegrin], mannose-binding lectin, and hMGL [human macrophage galactose- and N-acetylgalactosamine-specific C-type lectin]) bind N- and O-linked glycans on EBOV GP, leading to enhanced EBOV entry, although the details of how these interactions lead to virion internalization have yet to be studied. Cells lacking CLEC expression remain permissive for EBOV infection, providing evidence that CLEC-independent uptake mechanisms also occur. More recently appreciated is the role of cellular receptors that bind to PtdSer present in the viral envelope (reviewed in [7]). These PtdSer receptors include members of the T-cell immunoglobulin and mucin domain (TIM) family, TIM-1 and TIM-4, and protein complexes composed of Gas6 or Protein S and the TAM family of receptor tyrosine kinases, Tyro3, Axl, and Mer. Given that PtdSer is believed to be present on the surface of most, if not all, viral envelopes, it is not surprising that virion entry via virion-associated PtdSer/host PtdSer receptors interactions is not limited to filoviruses, but has recently been observed to mediate entry of a variety of enveloped viruses including flaviviruses, alphaviruses, and baculoviruses. While evidence suggests that TIM-1 can mediate virion internalization without cytoplasmic tail signaling [3,8], mechanistic details of TIM-1-dependent virion uptake are not currently established. In contrast, virus/TAM family receptor interactions trigger a signaling cascade that dampens the cell’s innate immune response, increasing the target cell permissivity [9]. Interestingly, enveloped virus interactions with CLECs may also immunomodulate immune responses of virus-infected DCs [10]. The relative importance of these various cell surface interactions for EBOV entry and pathogenesis, regardless of whether they bind GP glycans or envelope lipids, remains to be characterized in vivo.

Ebola virions are thought to be internalized primarily through macropinocytosis (Fig 3B), although other routes of uptake have also been reported [11–14]. The mechanism triggering EBOV uptake remains unknown; however, we and others have shown that PtdSer receptor-dependent internalization of viral particles does not require the presence of a viral glycoprotein on the particle [4,15]. Further, virion binding to CLECs or PtdSer receptors has not been shown to directly trigger macropinocytosis, so elucidation of the mechanism eliciting filovirion macropinocytosis is still needed. Another piece of the virion internalization puzzle that remains unsolved is how vesicular stomatitis virus (VSV) pseudovirions that are pseudotyped with EBOV GP internalize via macropinocytosis, whereas VSV containing its native G glycoprotein enters cells through clathrin-coated pits [16,17]. One possible explanation for this apparent disparity is that VSV displaying its native G glycoprotein interacts directly with its recently identified cellular receptor, the ubiquitous LDL receptor [18]. In contrast, the EBOV GP on pseudotyped VSV does not strongly interact with any cell surface receptors and EBOV instead utilizes less specific, lower affinity internalization mechanisms, such as PtdSer/PtdSer receptor and/or glycan/lectin interactions. Potentially, it is through these latter interactions that EBOV macropinocytosis occurs.

## Processing and Trafficking

Ebola virions internalize into early endosomes and subsequently traffic to the late endosome/lysosome in a Rab5 and Rab7 GTPase-dependent manner [19]. Within the endosome, low-pH-dependent proteases remove the heavily glycosylated MLD and glycan cap from GP1, resulting in a 17- to 19-kDa protein (Fig 3C) [20,21]. Cathepsins L and B were initially identified as the proteases essential for EBOV GP processing and their cleavage sites within EBOV GP have been mapped [22,23]. However, bacterial thermolysin and proteases present in Vero E6 cells and mouse embryonic fibroblasts can effectively substitute for these cathepsins [20,24]. Proteolytic processing of GP is necessary for exposure of the GP1 receptor binding domain (RBD), but is insufficient to initiate virus fusion at 37°C [5,25]. However, at higher temperatures under low pH and/or mild reducing conditions, the 19-kDa form of the GP binds to liposomes, suggesting that this version of the trimer is in a fusion-ready state [26].

## Endolysosomal Receptor Binding

The exposed RBD of the proteolytically primed GP1 binds to the late endosomal/lysosomal protein NPC1 (Fig 3D) [27,28]. This novel endosomal interaction is essential for subsequent filovirus/cell membrane fusion. Whether processed GP interaction with NPC1 directly triggers fusion or subsequent steps are needed remains unclear. Several groups have shown that additional endosomal proteolysis and/or reduction are required for EBOV fusion, but the chronology and endosomal location of these events have yet to be clarified [20,21,26,29].

EBOV membrane fusion events are thought to be similar to those described for other viral glycoproteins [30]. A hydrophobic fusion loop of GP2, normally buried beneath a neighboring GP1 monomer [5], becomes exposed by the fusion trigger (Fig 2 and Fig 3E). Low pH conditions are necessary for conformational changes within the fusion loop that promote fusion [31]. In the form of a fist-like structure, hydrophobic GP2 residues present at the tip of the fusion loop insert into the target membrane [32]. The GP2 trimer unwinds and refolds into a six-helix bundle in which the fusion loop and GP transmembrane domain meet [33]. The resulting fusion pore allows for release of the RNP into the cytoplasm and the start of virus replication (Fig 3F).

## Lessons from EBOV Entry Studies and Outstanding Questions

To date, investigation of EBOV entry has led to three paradigm-shifting insights. First, the discovery of PtdSer receptor-mediated entry for not only filoviruses but also for a variety of other enveloped viruses has helped to mechanistically elucidate the broad tropism of some enveloped viruses. Second, recognition of the low pH-dependent endosomal proteolytic processing of EBOV GP identified a novel low-pH-dependent mechanism that has now been shown to be required for a number of enveloped viruses. Third, identifying an endosomal receptor for filoviruses altered the understanding of potential locations of virus/receptor interactions. While this latter observation was initially made for filoviruses, recent studies have shown that lysosomal-associated membrane protein 1 (LAMP1) is a lysosomal receptor for Lassa virus, suggesting this endosomal mechanism of fusion control may be broader than previously appreciated [34].

Despite the progress over the past five years in understanding EBOV entry, many critical questions remain unanswered. For instance, how does PtdSer become enriched on the outer leaflet of enveloped virus membranes? Do virion interactions with CLECs, TIM proteins, or TAM/Gas6 complexes mediate direct internalization of virions into endosomes? If TIM proteins do directly mediate virus internalization, mechanistically, how is this accomplished since some TIM molecules are not thought to signal? Alternatively, do these receptors solely accumulate virions on the surface of cells? If so, is there a yet unidentified cell-surface receptor that is required for EBOV internalization? If PtdSer receptors do directly mediate EBOV internalization, do the different receptors mediate internalization through the same endosomal pathway and into the same endosomal compartment? For those filoviruses that do not require Cathepsin B and L, what endosomal proteases are responsible for their GP processing? What is the role of NPC1? Does NPC1 binding directly lead to EBOV fusion? If so, why are additional proteolysis events required and what protease(s) in which vesicular compartment mediate this second processing step? Finally, in terms of the big picture, what selective advantages are there for enveloped viruses such as filoviruses to use relatively non-specific, low-affinity mechanisms for internalization? Certainly, a growing body of evidence suggests that there must be advantages. These might include a breadth of tropism that would otherwise not be available to enveloped viruses. Further, using these entry mechanisms protects critical RBD and fusion residues from neutralizing antibodies by limiting extracellular exposure of these GP elements; in the current entry model for filoviruses, these sequences are solely exposed late within the endosomal/lysosomal compartments. A better understanding of these paradigm-shifting findings could provide additional drug targets to complement the current repertoire of Ebola virus antivirals in development. No doubt, the continued pursuit of answers to these questions by a number of groups will provide significant insights into both EBOV and, more broadly, virus biology.
